# EHMTI-0164. Concurrent headache and sleep problems: findings from the Danish national health survey

**DOI:** 10.1186/1129-2377-15-S1-B19

**Published:** 2014-09-18

**Authors:** N Lund, M Westergaard, M Barloese, C Glümer, R Jensen

**Affiliations:** 1 Department of Neurology Glostrup Hospital University of Copenhagen, Danish Headache Center, Glostrup, Denmark; 2 Capital Region of Denmark, Research Center for Prevention and Health, Glostrup, Denmark

## Introduction

Headache and sleep problems are highly prevalent in the general population but no epidemiological study has previously characterized people who report having both problems at the same time.

## Aims

To determine the prevalence of concurrent headache and sleep problems (HSP) and, for the first time, characterize respondents with HSP in terms of demographic, socioeconomic and lifestyle factors, psychological symptoms, and quality of life.

## Methods

129,150 randomly selected individuals were invited to participate in the Danish National Health Survey 2010. Respondents were asked about headache, sleep problems, psychological problems, and health-related lifestyle. Stress was measured using the Perceived Stress Scale. Quality of life was measured using SF-12. Prevalence proportions were adjusted for stratified sampling and non-response.

## Results

Of the 68,518 respondents (53.1% response), 17.3% reported only headache, 21.1% only sleep problems, and 18.1% HSP. HSP prevalence was higher among women and the middle-aged. HSP was inversely related to socioeconomic position. The group with HSP had significantly higher proportions of smokers, obese, and physically inactive, compared to the group without HSP (p<0.0001). Almost half (43.9 %) were in the highest stress quintile, 18.6 % reported depression, and 15.9 % anxiety. They also had the lowest quality of life scores.

## Conclusions

As the first large population-based study, we found that HSP is highly prevalent and significantly lowers quality of life. Lifestyle modification and screening for psychological problems may play an important role in prevention and management. The high prevalence of concurrent disorders suggests a common pathophysiological mechanism.

No conflict of interest.

